# Impact of driving cessation on health-related quality of life trajectories

**DOI:** 10.1186/s12955-024-02231-4

**Published:** 2024-02-01

**Authors:** Thelma J. Mielenz, Haomiao Jia, Carolyn DiGuiseppi, Lisa J. Molnar, David Strogatz, Linda L. Hill, Howard F. Andrews, David W. Eby, Vanya C. Jones, Guohua Li

**Affiliations:** 1 Department of Epidemiology, Mailman School of Public Health, Columbia University, New York, NY 10032 USA; 2 Center for Injury Science and Prevention, Columbia University Irving Medical Center, New York, NY 10032 USA; 3 Department of Biostatistics, Mailman School of Public Health, Columbia University, New York, NY 10032 USA; 4 Department of Epidemiology, Colorado School of Public Health, University of Colorado Anschutz Medical Campus, Aurora, CO 80045 USA; 5 University of Michigan Transportation Research Institute, Ann Arbor, MI 48109 USA; 6 Bassett Research Institute, Cooperstown, NY 13326 USA; 7 School of Public Health, University of California San Diego, La Jolla, CA 92093 USA; 8 Department of Psychiatry, Vagelos College of Physicians and Surgeons, Columbia University, New York, NY 10032 USA; 9 Department of Health, Behavior and Society, Johns Hopkins Bloomberg School of Public Health, Baltimore, MD 21205 USA; 10 Department of Anesthesiology, Vagelos College of Physicians and Surgeons, Columbia University, New York, NY 10032 USA

**Keywords:** Health-related quality of life and driving cessation

## Abstract

**Background:**

Trajectories of health-related quality of life (HRQoL) after driving cessation (DC) are thought to decline steeply, but for some, HRQoL may improve after DC. Our objective is to examine trajectories of HRQoL for individuals before and after DC. We hypothesize that for urban drivers, volunteers and those who access alternative transportation participants’ health may remain unchanged or improve.

**Methods:**

This study uses data from the AAA Longitudinal Research on Aging Drivers (LongROAD) study, a prospective cohort of 2,990 older drivers (ages 65–79 at enrollment). The LongROAD study is a five-year multisite study and data collection ended October 31, 2022. Participants were recruited using a convenience sample from the health centers roster. The number of participants approached were 40,806 with 7.3% enrolling in the study. Sixty-one participants stopped driving permanently by year five and had data before and after DC. The PROMIS®-29 Adult Profile was utilized and includes: 1) Depression, 2) Anxiety, 3) Ability to Participate in Social Roles and Activities, 4) Physical Function, 5) Fatigue, 6) Pain Interference, 7) Sleep Disturbance, and 8) Numeric Pain Rating Scale.

Adjusted (age, education and gender) individual growth models with 2989 participants with up to six observations from baseline to year 5 in the models (ranging from *n* = 15,041 to 15,300) were utilized.

**Results:**

Ability to participate in social roles and activities after DC improved overall. For those who volunteered, social roles and activities declined not supporting our hypothesis. For those who accessed alternative transportation, fatigue had an initial large increase immediately following DC thus not supporting our hypothesis. Urban residents had worse function and more symptoms after DC compared to rural residents (not supporting our hypothesis) except for social roles and activities that declined steeply (supporting our hypothesis).

**Conclusions:**

Educating older adults that utilizing alternative transportation may cause initial fatigue after DC is recommended. Accessing alternative transportation to maintain social roles and activities is paramount for rural older adults after DC especially for older adults who like to volunteer.

## Introduction

Driving cessation (DC) has historically been associated with a decrease in social, physical and mental health outcomes in older adults, but these declines are mitigated over time [1, 2]. Research is emerging that health-related quality of life (HRQoL) may actually improve after DC due to increased time with family members or increased physical activity from using alternative transportation [3–7]. Individual HRQoL changes after DC may be obscured in aggregated data; individual-level modeling methods are therefore preferred [2, 7–9].

The goal is to examine the trajectories of eight domains of HRQoL for individuals before and after DC. Based on current research, we hypothesize that for different subgroups (i.e., urban drivers, volunteers and those that access to alternative transportation) participants’ social and physical health will improve [3–7].

## Methods

### Participants, design and procedures

The AAA Longitudinal Research on Aging Drivers (LongROAD) cohort is a multisite prospective cohort of 2,990 older drivers [10]. Participants aged 65–79 years were recruited between July 2015 and March 2017 from: Ann Arbor, MI; Baltimore, MD; Cooperstown, NY; Denver, CO; and San Diego, CA. Key inclusion criteria for the study, include: 1) having a valid driver’s license, 2) staying in their current location for another five years, and 3) driving on average at least one time a week [10]. The LongROAD study is a five year study and data collection was completed October 31, 2022. The baseline visit and Year 2 were conducted in-person and Years 1 and 3 by telephone. Due to the COVID-19 pandemic, 33% of follow-up visits in Year 4 and 35% in Year 5 were conducted in-person with the rest conducted by a telephone interview.

## Measures

### Personal characteristics

Of the 2990 participants, 42% were in the 65–69 age category at baseline, 86% were non-Hispanic white, 53% were female, 63% were married, 41% had an advanced degree, 13% lived in rural areas (rural–urban commuting area codes: 4 and higher micropolitan/small town/rural), 54% volunteered and 11% accessed transportation options other than driving themselves, including public, on-demand, micro-mobility and friends/family [10].

### Exposure

DC was operationalized as those who voluntarily or involuntarily stopped driving permanently, as determined by: 1) questions about driving status at each annual follow-up visit, 2) participants notifying the study team that they stopped driving, and 3) participants’ driving activity stopped based on objective driving data recorded from their vehicle [11]. If there was no activity for at least 30 days, then the study team reached out to the participants to identify their current driving status. Seventy-three participants stopped driving during the follow-up period. One person started driving again and thus was excluded from the analysis for a total of 2989 in the models. Sixty-nine participants who stopped driving were determined by questions about driving status at each annual follow-up visit. Three participants were determined by the other two methods. The year since DC is defined as the interview year minus the year of DC.

### Primary outcomes

The outcomes were assessed before and after DC at their annual visits. Patient-Reported Outcomes Measurement Information System® or PROMIS®-29 Adult Profile (found to have construct validity and be reliable across three standard deviations) includes: 1) v1.0 (version 1.0)- Depression-4a (four items), 2) v1.0—Anxiety-4a, 3) v2.0 – Ability to Participate in Social Roles and Activities-4a, 4) Physical Function-4a, 5) v1.0-Fatigue-4a, 6) v1.0-Pain Intererence-4a, 7) v1.0-Sleep Disturbance-4a, and 8) Numeric Rating Scale v1.0 -Pain Intensity 1a [12].

### Statistical analyses

A trajectory of each outcome measure was examined with an individual growth model, a type of linear mixed model with repeated measures of 2989 with the observations in the models ranging from 15,041 to 15300 [13]. The main independent variables were (1) year since the baseline (0, 1, …,5), (2) a binary indicator of DC (0, 1), and (3) years since DC (i.e., a segmented regression model) [14]. The DC variable was used to measure change in outcome (i.e., level) immediately after DC. The years since DC variable was used to measure change in outcome trend (i.e., slope) after DC. We estimated adjusted (age, gender and education) trajectories of each outcome based on the individual growth model.

To estimate and compare trajectories by subgroups (accessed transportation, volunteer, and rural–urban areas), we included the subgroup indicator, as well as interaction terms of subgroup indictor with the DC variable and year since DC variable in the model. We also included age, gender and education in the model as covariates to control for bias between different subgroups.

## Results

### Response and participation rates

The follow-up for years one through five are respectively: 96.5%, 90.8%, 85.2%, 74.4% and 68%. Sixty-one participants, out of the 72 who reported permanent DC, stopped driving after baseline and before year 5 follow-up. There was no record of the outcome if the participant died or withdrew from the study.

### Characteristics of participants with driving cessation

Of the 61 participants who stopped driving, 30% were in the 65–69 age category, 80% were non-Hispanic white, 48% were female, 61% were married, 31% had an advanced degree, 15% lived in rural areas, 59% volunteered and 13% had access to transportation. Sixty-seven percent voluntarily stopped driving. The main reasons for stopping driving included: 1) specific medical condition – 32.76%, 2) 2) other reasons (e.g., license was suspended, car not working, suggested to stop driving by eye doctor etc.)– 20.69%, 3) problems with vision -13.79% 4) problems with mobility – 8.62%, 5) financial reasons – 8.62%, 6) loss of confidence in driving – 5.17%, 7) medications that may affect driving – 3.45%, 8) problems with memory – 3.45%, 9) got in a crash – 1.72% and 10) just don’t want to drive anymore – 1.72%.

### Trajectories

**Fig. 1:**
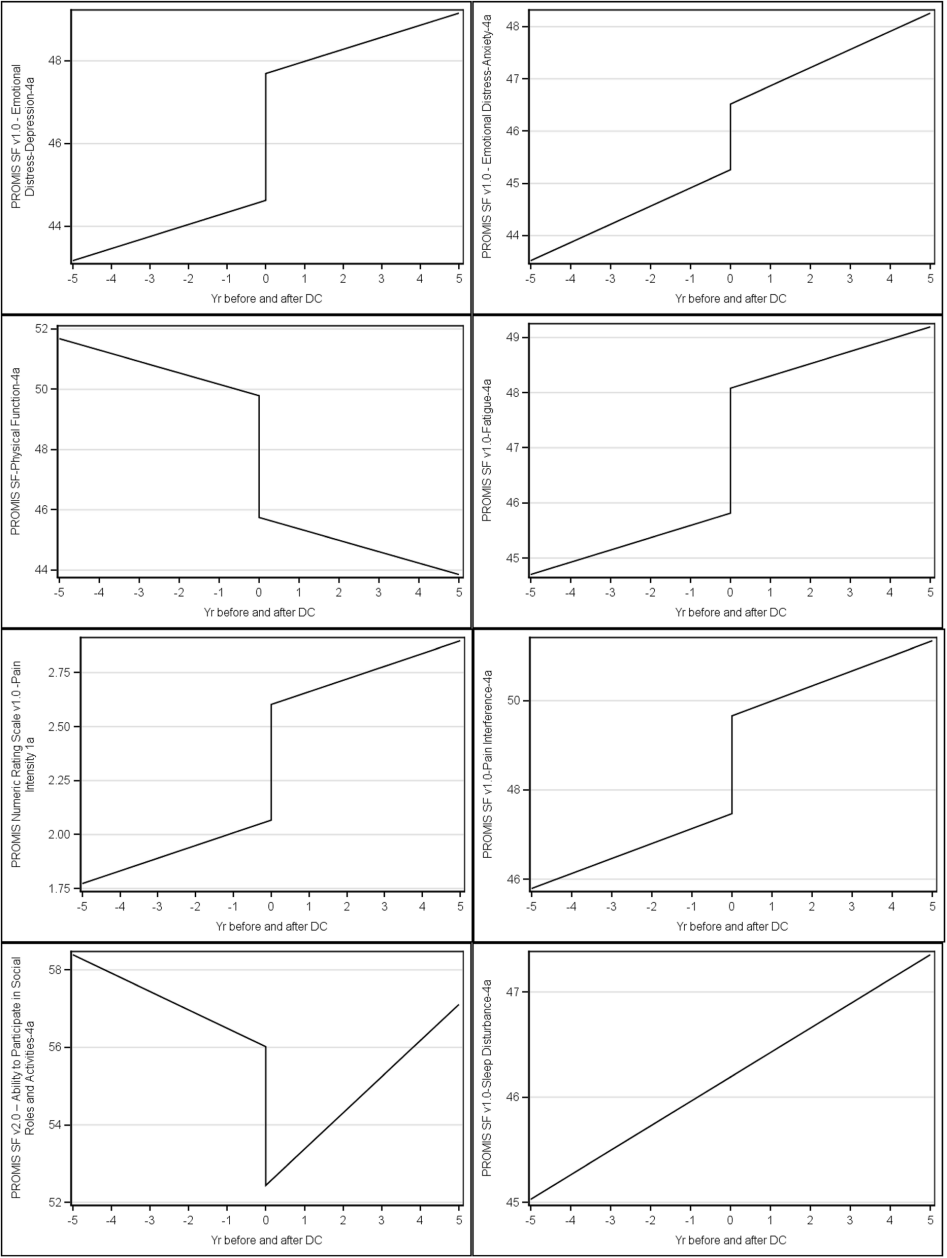
Effects of driving cessation on health-related quality of life

Depression (β 3.07), anxiety (β 1.26), physical function (β -4.04), fatigue (β 2.27), pain interference (β 2.19) and pain intensity (β 0.54) all worsened immediately after DC, but the slope of decline was no different than during the pre-DC period (Fig. 1 and Table 1). In contrast, the Ability to Participate in Social Roles and Activities declined immediately after DC (β -3.58) but then increased with a steep slope (β 1.41). Sleep disturbance did not change before or after DC and continued to increase at the same rate.

**Table 1:** Results of individual growth curve models examining the effects of driving cessation on health-related quality of life

Variables	Estimate	SE	95% CI	*p*-value
PROMIS SF v1.0—Emotional Distress-Depression-4a				
Driving cessation	3.07	0.50	2.09, 3.99	< .0001
Time since driving cessation^a^	0.00	–	–	–
PROMIS SF v1.0—Emotional Distress-Anxiety-4a				
Driving cessation	1.26	0.56	0.16, 2.30	0.0245
Time since driving cessation^a^	0.00	–	–	–
PROMIS SF v2.0 – Ability to Participate in Social Roles and Activities-4a				
Driving cessation	-3.58	0.72	-4.98, -2.14	< .0001
Time since driving cessation	1.41	0.54	0.36, 2.41	0.0086
PROMIS SF v1.0-Fatigue-4a				
Driving cessation	2.27	0.68	0.93, 3.54	0.0009
Time since driving cessation^a^	0.00	–	–	–
PROMIS SF-Physical Function-4a				
Driving cessation	-4.04	0.54	-5.09, -3.05	< .0001
Time since driving cessation^a^	0.00	–	–	–
PROMIS SF v1.0-Pain Interference-4a				
Driving cessation	2.19	0.74	0.74,3.56	0.003
Time since driving cessation^a^	0.00	–	–	–
PROMIS SF v1.0-Sleep Disturbance-4a				
Driving cessation	0.00	–	–	–
Time since driving cessation^a^	0.00	–	–	–
PROMIS Numeric Rating Scale v1.0 -Pain Intensity 1a				
Driving cessation	0.54	0.23	0.09,0.96	0.0181
Time since driving cessation^a^	0.00	–	–	–

^a^This term was excluded from the model because it was not statistically significant

**Fig. 2:**
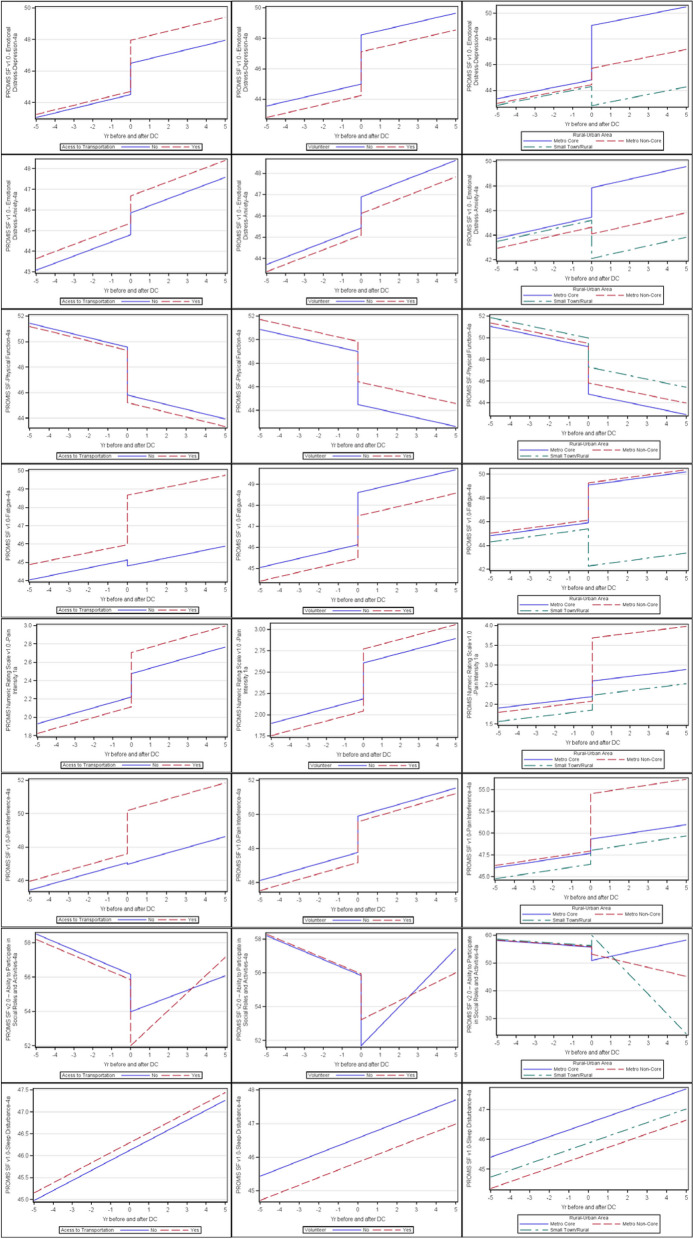
Stratified effects of driving cessation on health-related quality of life

Figure 2 visualizes the trajectories stratified by accessing transportation, volunteer status and rural–urban area after controlling for age, gender, and education. For accessing transportation, the slope declines at the same trend for depression, pain symptoms, sleep disturbance and anxiety; and improves for social roles and activities, particularly for participants with post-DC utilization of transportation. Those accessing transportation had a large immediate increase in fatigue after DC but the slope then continues with the same trend. Those participants who volunteer have fewer symptoms and better function before and after DC except participation in social roles and activities, which improved less for volunteers than for non-volunteers after DC. Similarly, participants with rural residence had better function and fewer symptoms before and after DC except for social roles and activities, which declined steeply compared to their more urban counterparts.

## Discussion

The ability to participate in social roles and activities appeared to increase after DC, overall and in those accessing alternative transportation and non-volunteers, which may be due to increased interaction with family and friends [2–6]. Non-volunteers improving relative to volunteers was counterintuitive. This may be due to volunteers not accessing transportation to their volunteer activities which can be an integral part of their social roles and activities. Future studies should look at whether participants maintained their volunteering over time. In contrast, ability to participate dropped steeply for those in rural areas, where DC was considered at a priori to have greater potential impact [15]. Interestingly, social roles and activities declined the most for rural residence which may be due to either the lack of accessing transportation or the lack friends and family living nearby.

Edwards et al. (2009) reported significant declines in health among former drivers, including social and physical health, physical performance and the greatest decline in general health [1]. However, Edwards et al.’s analysis included former drivers at baseline; thus, not all of the participants were followed prospectively through their transition to DC. In this LongROAD study, we were able to address these limitations as well as assess the health outcomes at time since driving cessation to see if the trajectories differ at a further time period since DC [1, 2]. Our measure of DC is precise and current. Heterogeneity in the effects on DC were addressed in only one previous study that used a cluster analysis, but they were unable to address DC that accounted for time [2]. We did not adjust for vision or cognition because the goal of this study was to evaluate the change in HRQoL; adjusting for any health-related variables would have attenuated any change in HRQoL.

## Conclusion

Participation in social roles and activities can increase after DC with utilization of alternative forms of transportation. Decreasing social roles and activities after DC are especially steep in rural areas. Efforts should be made to increase utilization of alternative forms of transportation in rural areas including public, on-demand, micro-mobility and friends/family.

## Data Availability

Data sharing is restricted due to limitations within the consent forms.

## Abbreviations

DC: Driving cessation
HRQoL: Health-related quality of life
LongROAD: Longitudinal Research on Aging Drivers
MI: Michigan
MD: Maryland
NY: New York
CO: Colorado
CA: California
PROMIS®: Patient-Reported Outcomes Measurement Information System®
